# A descriptive study of sports chiropractors with an International Chiropractic Sport Science Practitioner qualification: a cross-sectional survey

**DOI:** 10.1186/s12998-021-00405-1

**Published:** 2021-12-13

**Authors:** Luke Nelson, Henry Pollard, Rick Ames, Brett Jarosz, Pete Garbutt, Cliff Da Costa

**Affiliations:** 1 Health and High Performance, 437 Belmore Rd, Mont Albert North, VIC 3129 Australia; 2 CQ University, Sydney, Australia; 3 RMIT University, Melbourne, Australia; 4 Optimize Sports Chiropractic, South Yarra, Australia; 5 Enhance Healthcare, Canberra, Australia

**Keywords:** Sports chiropractic, Manipulation, Multimodal, ICSSP, Sports sciences, Cross-sectional survey

## Abstract

**Background:**

This paper describes the education and case management profile of sports chiropractors with the Federation of International Sports Chiropractors (FICS) postgraduate qualification: International Chiropractic Sport Science Practitioner (ICSSP). The ICSSP is the predominant international sports chiropractic qualification.

**Methods:**

A cross-sectional survey, carried out between 22/10/2014 and 22/12/2014,was utilized with a 39-item web-based survey examining practitioner, practice and clinical management characteristics, and was distributed via email to all sports chiropractors who held an ICSSP qualification (*n* = 240) in 2014.

**Results:**

The survey response rate was 64% (*n* = 154). 36% of chiropractors were aged between 31 and 40 years, just over three quarters were male, and 27% had been in practice for 5–10 years. The majority of respondents were based in North America. All sports chiropractors surveyed reported treating neuromusculoskeletal conditions outside of the spine. 91% utilized a multimodal approach in most of their treatments, prescribing rehabilitative exercises in 76% of consultations. Almost 64% of respondents reported current treatment of professional athletes, and 78% reported current treatment of semi-professional athletes, whilst the vast majority of those surveyed endorsed past treatment of professional (91%) and semi-professional (95%) athletes. All respondents reported referring to a range of conventional and allied health providers.

**Conclusions:**

This study of ICSSP-qualified sports chiropractors describes a small but well-educated workforce treating high-level athletes, managing a wide range of spine and non-spinal neuromusculoskeletal conditions, utilising multimodal approaches (including active and passive strategies), and referring to and co-managing with other health practitioners.

**Supplementary Information:**

The online version contains supplementary material available at 10.1186/s12998-021-00405-1.

## Background

The International Federation of Sports Chiropractic/Fédération Internationale de Chiropratique du Sport (FICS), founded in 1987, is comprised of national chiropractic sports councils worldwide and has affiliations with international organizations within the chiropractic profession and the world of sports. FICS has created a post-professional qualification program (the International Chiropractic Sport Science Practitioner [ICSSP]) for practitioners working at sporting events that consists of a minimum of an online educational component, a hands-on component, and a field-based experience requirement [1].

Over the past 30 years, the sports health care team has evolved to include speciality groups (medicine, physiotherapy, sports sciences, nutrition, podiatry and psychology) [2–4]. For many years amateur, professional, and now Olympic teams have utilised chiropractic care [5, 6]. It is the authors’ anecdotal experience that the inclusion of chiropractors within sports teams has historically been at the insistence of athletes rather than with the acceptance of others in the sports medicine team. However, many athletes have been denied access to chiropractic care even when they request it. What has changed in recent years is that a growing number of sports chiropractors are fully integrated into many sports medicine teams, up to and including major national and international games [6, 7]. At the 2010 Winter Olympic Games in Vancouver, the 2012 Summer Olympic Games in London, the 2016 Summer Olympic Games in Rio De Janeiro, and the 2020 Summer Olympic Games in Tokyo, sports chiropractors were included in the Olympic Village Polyclinic: the multi-disciplinary facility that offers health care and medical services to Olympic athletes, officials, and staff.

Chiropractors are often considered to be uni-modal practitioners with limited regard for orthodox medical approaches [3, 8]. However the literature indicates that chiropractors are not limited to a manipulation-only approach, with the majority of treatments being multi-modal [9]. In addition, a recent survey by Adams et al. found that nearly half of the surveyed Australian general chiropractors who treat athletes or sportspeople ‘often’ were more likely to use a multimodal approach to management compared to their colleagues who did not ‘often’ treat athletes or sportspeople [10].

Whilst previous studies have examined sports chiropractors in select countries [10–12], our study aimed to describe the practice characteristics of sports chiropractors who hold the ICSSP certification. An online questionnaire format was used to assess the educational and practice characteristics of ICSSP-qualified sports chiropractors. The survey specifically aimed to determine the general demographics of this group, the conditions they treat, the modalities and adjuncts they use, their involvement with high-level athletes, and their integration with other health professionals.

## Methods

This study was approved by the RMIT University, SEH College Human Ethics Advisory Network (ASEHAPP 52-14 AMES), and all respondents consented to their de-identified responses being utilized. The design consisted of a cross-sectional self-report web survey of sports chiropractors in the FICS organisation that held either of the FICS qualifications [see Additional file 1].

We included all those with an International Chiropractic Sport Science Practitioner (ICSSP) or the precedent qualification International Chiropractic Sport Science Diploma (ICCSD). Both courses had the same requirements despite the name change to the qualification.

Permission was granted from the FICS executive, to email the 39-item web-based “SoSci Survey” [13] to the 240 chiropractors with a FICS qualification on their database (2015). Three emails were sent over two months encouraging participation, between 22/10/2014 and 22/12/2014. Descriptive statistics were performed by SPSS to summarise the responses in terms of frequencies, percentages, 95% confidence intervals (CIs), means and standard deviations, as appropriate.

## Results

A total of 154 participants, out of 240 eligible ICCSP holders completed the survey, representing a 64% response rate. According to the survey data, the typical sports chiropractor was male (78% of respondents, 95% CI, 70.7–83.8%, Table 1), aged 31–40 (36%, 95% CI, 29.2–44.2%), and had accumulated 5–10 years of clinical practice experience (27%, 95% CI, 20.9 to 34.8). Regarding the geographical location of respondents, the majority practiced in North America (37%, 95% CI, 29.8–44.9%, Table 1), with Europe (27%, 95% CI, 20.9–34.8%) and Oceania (25%, 95% CI, 19.1–32.7%) also well represented.

**Table 1:** Demographics

Age group	N	%	95% CI
Male	120	77.9	70.7 to 83.8%
Female	34	22.1	16.3 to 29.3%
*Age group*			
21–30	27	17.5	12.3 to 24.3
31–40	56	36.4	29.2 to 44.2
41–50	33	21.4	15.7 to 28.6
51–60	34	22.1	16.3 to 29.3
61–70	4	2.6	1.0 to 6.5
*Years in practice*			
0–5	28	18.2	12.9 to 25.0
5–10	42	27.3	20.9 to 34.8
10–20	38	24.7	18.5 to 32.1
20–30	35	22.7	16.8 to 30.0
30 +	11	7.1	4.0 to 12.3
*Location of practice*			
Asia	5	3.3	1.4 to 7.4
Africa	9	5.8	3.1 to 10.7
Europe	42	27.3	20.9 to 34.8
North America	57	37.0	29.8 to 44.9
South America	2	1.3	0.4 to 4.6
Oceania	39	25.3	19.1 to 32.7

In addition to completing their ICSSP, 32% (95% CI, 25.0–39.5%) of sports chiropractors surveyed reported completion of a post-graduate certificate relevant to sports chiropractic. Furthermore, 23% (95% CI, 17.4–30.7%) endorsed completion of a post-graduate diploma, 17% (95% CI, 11.8–23.6%) a Masters, and 3% (95% CI, 1.0–6.5%) a PhD in the field of sports chiropractic.

An overwhelming majority of respondent chiropractors (96%, 95% CI, 90.9–97.8%) indicated reading health care related research, averaging 2.5 (95% CI, 2.0–3.0%) hours of weekly reading of articles related to sports chiropractic.

When it comes to working with sporting teams, 20% (95% CI, 14.6–27.2%) reported that they were currently working with a sports team full time. Treatment of professional and semi-professional athletes was common, with 64% (95% CI, 55.8–70.8%) of respondents reporting current treatment of professional athletes and 78% (95% CI, 70.7–83.8%) reporting treatment of semi-professional athletes. The vast majority of those surveyed endorsed having treated professional (91%, 95% CI, 85.3–94.5) and semi-professional (95%, 95% CI, 90.1–97.3) athletes in the past. In regards to Olympic level athletes, 38% (95% CI, 31.0–46.2%) reported current treatment of this athlete population and 64% (95% CI, 55.8–70.8%) reported having treated Olympic level athletes in the past.

Manipulative therapy was a commonly utilized modality by sports chiropractors, with all those surveyed utilizing spinal manipulation (95% CI, 97.6–100.0%), and 99% (95% CI, 96.4–99.9%) utilizing peripheral joint manipulation.

Most chiropractors endorsed a multimodal approach in their practice, with 91% (95% CI, 85.3–94.5%) surveyed using multiple modalities in most of their treatments. Other commonly used techniques include soft tissue therapy (97%, 95% CI, 93.5–99.0%), mobilisation (93%, 95% CI, 88.5–96.4%), kinesiotaping (91%, 95% CI, 85.3–94.5%), low force techniques (90%, 95% CI, 84.6–94.0%) and instrument assisted soft tissue therapy (83%, 95% CI, 76.4–88.2%). Less commonly used modalities include rigid taping (67%, 95% CI, 59.1–73.8%), physical therapeutics (58%, 95% CI, 49.9–65.3%) and dry needling (38%, 95% CI, 30.4–45.5%) (Table 2).

**Table 2:** Treatment modality

Treatment modality	N (154 respondents)	%	95% CI
Spinal manipulation	154	100	97.6 to 100
Peripheral manipulation	153	99.4	96.4 to 99.9
Mobilisation	144	93.5	88.5 to 96.4
Low force techniques	139	90.3	84.6 to 94.0
Soft tissue therapy	150	97.4	93.5 to 99.0
Dry needling	58	37.7	30.4 to 45.5
Instrument assisted soft tissue (IASTM)	128	83.1	76.4 to 88.2
Rigid taping	103	66.9	59.1 to 73.8
Kinesiotaping	140	90.9	85.3 to 94.5
Physical therapeutics	89	57.8	49.9 to 65.3

All of the sports chiropractors surveyed treat neuromusculoskeletal conditions (conditions involving nerves, muscles, soft tissue, and bones) outside of the spine, with 37% (95% CI, 34.3–40.1%) of patients presenting to these chiropractors with a non-spinal musculoskeletal primary complaint.

In addition to passive therapies, sports chiropractors also prescribe rehabilitative exercises on an average of 76% (95% CI, 72.1–79.9%) of all patient visits ergonomic advice 69% (95% CI, 63.6–72.8%) with nutritional advice less commonly prescribed to patients at 40% (95% CI, 34.9–44.5%) of all patient visits.

All of the sports chiropractors surveyed refer patients to other health practitioners, with 64% (95% CI, 55.8–70.8%) stating they often do so. The most commonly referred to health practitioners were orthopaedic surgeons (79%, 95% CI, 71.4–84.3%), massage therapists/myotherapists (77%, 95% CI, 69.3%), general medical practitioners (71%, 95% CI, 63.8–78.0%), physiotherapists/physical therapists (62%, 95% CI, 54.5–69.6%) and podiatrists (50%, 95% CI, 42.2–57.8%).

The majority (92%, 95% CI, 89.1–95.0%) of sports chiropractors co-manage with other health practitioners with 58% (95% CI, 50.6–66.0%) often doing so. Sports chiropractors most commonly co-manage patients with physiotherapists/physical therapists (60%, 95% CI, 51.9–67.2%), massage therapists (55%, 95% CI, 46.7–62.2%), orthopaedic surgeons (45%, 95% CI, 37.2–52.7%), general medical practitioners (45%, 95% CI, 37.2–52.7%), strength and conditioning professionals (36%, 95% CI, 28.6–43.5%) and sports physicians (31%, 95% CI, 24.4–38.9%).

## Discussion

This is the first survey to describe the demographic and practice characteristics of FICS based sports chiropractors. Based on the responses to this survey, it appears that ICSSP-qualified sports chiropractors are working with elite and professional athletes in many countries. The gender distribution of respondents in our study (78% male), is similar to that in the general chiropractic profession from studies in Australia, Norway and the United States (62%–79% male) [9, 14, 15], which suggests that ICSSP-qualified sports chiropractors are not underrepresented by females.

General practice chiropractors have historically been perceived as uni-modal [8,] but a 2009 survey of the United States general chiropractic profession by Christensen et al.. revealed that more than three-quarters of general chiropractors use passive adjunctive care procedures including ice packs, trigger point therapy, braces, and electrical stimulation [9]. Further, a recent survey by Adams et al. indicated that nearly half of the surveyed Australian general chiropractors who treat athletes or sportspeople ‘often’ were more likely to use a multimodal approach to management more than their colleagues that did not treat athletes or sportspeople ‘often’ [10]. The higher utilization of a multi-modal approach to treatment in our sports chiropractors (91%, 95% CI, 85.3–94.5%), may suggest that those who complete their ICCSP are more likely to utilize a multimodal approach than general chiropractors. However, the Adams et al. study only examined Australian chiropractors, and therefore may not be generalizable to chiropractors in other countries. Manipulative therapies are commonly combined with soft tissue therapy (used by 97% of chiropractors, 95% CI, 93.5–99.0%), mobilisation (94%, 95% CI, 88.5–96.4%), kinesiotaping (90%, 95% CI, 85.3–94.5%), low force techniques (90%, 95% CI, 84.6–94.0%), instrument assisted soft tissue therapy (83%, 95% CI, 76.4–88.2%), rigid taping (67%, 95% CI, 59.1–73.8%), physical therapeutics (58%, 95% CI, 49.9–65.3%) and dry needling (38%, 95% CI, 30.4–45.5%).

In conjunction to passive modalities, rehabilitative exercises were prescribed by our sports chiropractors in 76% (95% CI, 72.1–79.9%) of visits. Comparatively, previous studies have shown general chiropractors prescribing rehabilitation on a less frequent basis at 31% of visits, suggesting those working with active populations maybe more likely to prescribe exercises [16, 17]. Despite a thorough literature search, we could not locate a comparative figure explaining exercise prescription behaviours for physiotherapists and sports physiotherapists.

Chiropractors are often labelled “spine only” due to reasons that range from self-promotion by chiropractors to misrepresentations by non-chiropractors [18–20]. Our survey reveals that sports chiropractors commonly treat both spinal and non-spinal neuromusculoskeletal conditions. All of the sports chiropractors in our study treat non-spinal neuromusculoskeletal conditions and 37% (95% CI, 34.3–40.1%) of their patients have a primary complaint that is non-spinal musculoskeletal.

This survey also provides information on the self-education of sports chiropractors who are sometimes labelled as not conforming to evidence- based practice [3, 21]. As with medical practitioners, allied health care practitioners and other complementary and alternative medicine practitioners, chiropractors also adhere to an evidence-informed practice [2, 22]. Our study shows that sports chiropractors frequently read research related to their field. On average, respondents spend an average 2.5 h (95% CI, 2.0–3.0%) per week reading 2.9 (95% CI, 2.4–3.4%) research articles. Our survey did not examine the quality of the literature being consumed, the information retention from this reading or how this information is implemented in practice. This implementation of evidence into practice is not unique to chiropractic and sports chiropractic. It is noteworthy that similar issues have been described in orthodox medical and allied health groups. Some have suggested that a considerable amount of management in sports medicine is not supported by evidence [23]. It is noteworthy that other health practitioners have a difficult time deciding between the research-based evidence, patient choice and their clinical views [22, 24, 25] so it should not be surprising that sports chiropractors are also afflicted by this challenge.

Sports chiropractors are increasingly being used by elite sporting teams: the current NFL season saw 41 team chiropractors working with all 32 teams, and chiropractic services were offered in the Summer Olympic Games polyclinic in 2008, 2012, 2016 and 2020. As our study reveals, the vast majority of ICSSP chiropractors surveyed have at some stage in their careers treated professional (91%, 95% CI, 85.3–94.5) and semi-professional (95%, 95% CI, 90.1–97.3) athletes, with 20% (95% CI, 14.6–27.2%) currently working with a sports team full time.

There are those that hold the view that chiropractors do not integrate well in a main-stream multi-disciplinary team, nor do they interact with other professionals on a regular basis, as was shown in Busse et al.’s survey of orthopaedic surgeons [21]. Whilst this may have been true historically, Christensen et al.’s 2015 study, reported an increase in chiropractic co-management with other professionals in 2014 compared to 1998, which suggests an increasingly integrated approach to patient care [9]. These historical perceptions of poor integration were not observed in our study. The sports chiropractors surveyed in our study refer (64% (95% CI, 55.8–70.8%) often doing so) and co-manage (58% (95% CI, 50.6–66.0%) often doing so) with other health professionals. This need to pursue the best practice, evidence-based multimodal care is evident in the sports chiropractors described in our sample, as they integrate their skill set with others in the sports medicine team [18].

A strength of this study is that it attracted a good response rate of 64%, which is likely to be representative of the FICS based sports chiropractors [26, 27]. Whilst it may be the largest international sports chiropractic group, FICS is not representative of all sports chiropractic groups, and that remains a limitation of the study.

Additionally, the survey was written in English and therefore limited to English speaking FICS chiropractors. However, despite the name FICS, the ICSSP/D qualification is only presented in English and therefore those who have completed it would be expected to be proficient in English.

This preliminary study described the activities of sports chiropractors. Future research could investigate different geographical, practice and practitioner characteristics, comparing sports chiropractors at different levels of involvement (local, state, national, international) as well as describing in detail the treatment outcomes obtained by sports chiropractors.

## Conclusions

This study of sports chiropractors describes a small but well-educated workforce, that treats high-level athletes, manages a wide range of spine and non-spinal neuromusculoskeletal conditions, utilises a multimodal approach (including active and passive strategies), and refers to and co-manages with other health practitioners.

## Supplementary Information

**Additional file 1:** Web survey questionnaire.

## Declarations

### Availability of data and materials

The Data set is available by request through the corresponding author.

## References

[1] ICSC Program Overview. https://ficssport/icsc-program-overview/. Accessed 12th Aug 2021.

[2] Miners AL, Degraauw C (2010). A survey of Fellows in the College of Chiropractic Sports Sciences (Canada): their intervention practices and intended therapeutic outcomes when treating athletes. J Can Chiropr Assoc.

[3] Pollard H, Hoskins W, McHardy A, Bonello R, Garbutt P, Swain M, Dragasevic G, Pribicevic M, Vitiello A (2007). Australian chiropractic sports medicine: half way there or living on a prayer?. Chiropr Osteopat.

[4] Thompson B, MacAuley D, McNally O, O'Neill S (2004). Defining the sports medicine specialist in the United Kingdom: a Delphi study. Br J Sports Med.

[5] Stump JL, Redwood D (2002). The use and role of sport chiropractors in the national football league: a short report. J Manipulative Physiol Ther.

[6] Nook DD, Nook EC, Nook BC (2016). Utilization of Chiropractic Care at the World Games 2013. J Manipulative Physiol Ther.

[7] Nook DD, Nook BC (2011). A report of the 2009 World Games injury surveillance of individuals who voluntarily used the International Federation of Sports Chiropractic delegation. J Manipulative Physiol Ther.

[8] Robson, S. Joint manipulation: is it safe? (2006). http://www.pponline.co.uk/encyc/joint-manipulation-is-it-safe-35852#. Accessed 22 Mar 2015

[9] Christensen M, Hyland J, Goertz CM, Kollasch M, Shotts B, Blumlein N, Johnson J, Day A, Smith M: Practice Analysis of Chiropractic 2015: a project report, survey analysis, and summary of chiropractic practice in the United States. National Board of Chiropractic Examiners. 2015.

[10] Adams J, Lauche R, de Luca K, Swain M, Peng W, Sibbritt D (2018). Prevalence and profile of Australian chiropractors treating athletes or sports people: a cross-sectional study. Complement Ther Med.

[11] Myburgh C, Andersen J, Bakkely N, Hermannsen J, Zuschlag M, Damgaard P, Boyle E (2021). The Danish sports chiropractic landscape: an exploration of practice characteristics and salient developmental issues. Chiropr Man Therap.

[12] Pucciarelli A, Randall N, Hayward M, Triantis J, Owen W, Swain M, de Luca K (2020). Sports chiropractors in Australia: a cross-sectional survey. J Can Chiropr Assoc.

[13] SoSci Survey. https://wwwsoscisurveyde/. Accessed 4th Nov 2021.

[14] Adams J, Lauche R, Peng W, Steel A, Moore C, Amorin-Woods LG, Sibbritt D (2017). A workforce survey of Australian chiropractic: the profile and practice features of a nationally representative sample of 2,005 chiropractors. BMC Complement Altern Med.

[15] Kvammen OC, Leboeuf-Yde C (2014). The chiropractic profession in Norway 2011. Chiropr Man Therap.

[16] Radovic S, Melvin GA, Gordon MS (2018). Clinician perspectives and practices regarding the use of exercise in the treatment of adolescent depression. J Sports Sci.

[17] Beliveau PJH, Wong JJ, Sutton DA, Simon NB, Bussieres AE, Mior SA, French SD (2017). The chiropractic profession: a scoping review of utilization rates, reasons for seeking care, patient profiles, and care provided. Chiropr Man Therap.

[18] Theberge N (2008). The integration of chiropractors into healthcare teams: a case study from sport medicine. Sociol Health Illn.

[19] Donovan J, Cassidy JD, Cancelliere C, Poulsen E, Stochkendahl MJ, Kilsgaard J, Blanchette MA, Hartvigsen J (2015). Beyond the spine: a new clinical research priority. J Can Chiropr Assoc.

[20] Hoskins W, Pollard H, Garbutt P (2009). How to select a chiropractor for the management of athletic conditions. Chiropr Osteopat.

[21] Busse JW, Jacobs C, Ngo T, Rodine R, Torrance D, Jim J, Kulkarni AV, Petrisor B, Drew B, Bhandari M: Attitudes toward chiropractic: a survey of North American orthopedic surgeons. Spine (Phila Pa 1976). 2009, 34(25):2818–2825. 10.1097/BRS.0b013e3181c1512f 19910864

[22] Hadley J, Hassan I, Khan KS (2008). Knowledge and beliefs concerning evidence-based practice amongst complementary and alternative medicine health care practitioners and allied health care professionals: a questionnaire survey. BMC Complement Altern Med.

[23] Orchard JW, Brukner PD (2005). Sport and exercise medicine in Australia. Med J Aust.

[24] Orchard J (2015). Translating evidence-based sports medicine into clinical practice: time to point the finger at doctors as much as at patients with respect to knee arthroscopy. Br J Sports Med.

[25] Ardern CL, Dupont G, Impellizzeri FM, O'Driscoll G, Reurink G, Lewin C, McCall A: Unravelling confusion in sports medicine and sports science practice: a systematic approach to using the best of research and practice-based evidence to make a quality decision. Br J Sports Med. 2017. 10.1136/bjsports-2016-097239 28818957

[26] Fincham JE (2008). Response rates and responsiveness for surveys, standards, and the Journal. Am J Pharm Educ.

[27] Berman DM, Tan LL, Cheng TL (2015). Surveys and response rates. Pediatr Rev.

