# Supplementary material for: A descriptive study of sports chiropractors with an International Chiropractic Sport Science Practitioner qualification: a cross-sectional survey
Source: Chiropr Man Therap. 2021 Dec 13;29:51. doi: 10.1186/s12998-021-00405-1 (PMC8667448; doi:10.1186/s12998-021-00405-1)
Supplement: Supplementary file 1 — Additional file 1: Web survey questionnaire. [file 12998_2021_405_MOESM1_ESM.pdf]

1. CONSENT

INVITATION TO PARTICIPATE IN A RESEARCH PROJECT

PARTICIPANT INFORMATION

Project Title: A descriptive study of Sports Chiropractors with an ICCSP qualification

Investigators:

Dr. Luke Nelson, B App Sci (Clin Sci.)/B Chiro Sci, ICSSD, PGrad.Dip.SportChiro, M.Sport.Sci, Email:luke@chirosolutions.com.au, Phone: +61395570251

Dr. Rick Ames, BSc, DC, FACCS, ICSSD, FACC. Email: rick.ames@rmit.edu.au Phone: +6139925 7394

Dr. Brett Jarosz, BAppSc(CompMed), MClinChiro, ICSSD, PGradDipSportsChiro, DACNB, FICC. Email: brett.jarosz@rmit.edu.au

Dr. Henry Pollard BSc, Grad DC, Grad Dip AppSc, MSportSc, PhD, ICSSD. Email: hpollard@optushome.com.au

Dr. Pete Garbutt B.Sc., M.Chiro., M.Chiro.Sports.Sci., ICSSD. Email: enhancehealthcare@iinet.net.au

Dr Cliff Da Costa, BSc, MSc, MS, PhD. Email: cliff.dacosta@rmit.edu.au. Phone: 9925 6114

Dear participant,

You are invited to participate in a research project being conducted by RMIT University. Please read this page carefully and be confident that you understand its contents before deciding whether to participate. If you have any questions about the project, please email Luke Nelson at luke@chirosolutionscom.au.

Who is involved in this research project? Why is it being conducted?

This study has been approved by the RMIT Human Research Ethics Committee, and also has the approval and support of FICS.

Dr’s Nelson, Ames, Jarosz, Pollard and Garbutt are Chiropractors that will all equally contribute to the writing of the completed study. Dr Cliff Da Costa is a statistician who will assist with the statistical analysis of the data in this study.

Why have you been approached?

You have been approached with this invitation as you currently hold an ICCSP/ICSSD qualification through FICS. No contact details have been passed onto the investigators, invitations have only been sent through FICS themselves.

What is the project about? What are the questions being addressed?

This project aims to look at how those that hold an ICCSP qualification practice. Only those that hold an ICCSP qualification can complete this survey. Currently there are approximately 240 Chiropractors with this qualification, the investigators are hoping that the majority of these complete the survey.

The primary research questions that will be examined in this study are:

- The general demographics of this group
- Are those that hold an ICCSP multi-modal in their treatment approach?
- Do they treat non spinal musculoskeletal conditions?
- Do they refer to other health practitioners?
- Do they co-manage patients with other health practitioners?
- Do they use evidence informed treatments?
- Do they regularly read research?
- Do they treat athletes involved in professional/semi professional sport?
- Do they treat athletes involved in Olympic teams?
- Do they regularly prescribe rehabilitation exercise?
- Do they prescribe ergonomic advice?
- Do they prescribe nutritional advice?

If I agree to participate, what will I be required to do?

If you agree to participate you simply need to complete the following questionnaire which will take approximately 5-7mins to complete.

What are the possible risks or disadvantages?

There are no perceived risks in participating in this study.

What are the benefits associated with participation?

Whilst there is no direct benefit to you as a result of your participation, the data revealed in this study may help FICS’ involvement in future sporting events, and also allow them to plan relevant continuing education.

What will happen to the information I provide?

The information that you provide will be anonymous, meaning the data that you provide cannot be identified at any stage of this research project

The results will be published in a yet to be determined journal, and also be available to all participants in a future FICS newsletter.

The research data will be kept securely at RMIT for 5 years after publication, before being destroyed. Whereas the final research paper will remain online.

Because of the nature of data collection, we are not obtaining written informed consent from you. Instead, we assume that you have given consent by your completion and return of the materials.

Security of the website

Users should be aware that the World Wide Web is an insecure public network that gives rise to the potential risk that a user's transactions are being viewed, intercepted or modified by third parties or that data which the user downloads may contain computer viruses or other defects.

Security of the data

This project will use an external site to create, collect and analyse data collected in a survey format. The site we are using is https://www.soscisurvey.de. If you agree to participate in this survey, the responses you provide to the survey will be stored on a host server that is used by https://www.soscisurvey.de. No personal information will be collected in the survey so none will be stored as data. Once we have completed our data collection and analysis, we will import the data we collect to the RMIT server where it will be stored securely for five (5) years. The data on the https://www.soscisurvey.de host server will then be deleted and expunged.

What are my rights as a participant?

• The right to withdraw from participation at any time

• The right to request that any recording cease

• The right to have any unprocessed data withdrawn and destroyed, provided it can be reliably identified, and provided that so doing does not increase the risk for the participant.

• The right to have any questions answered at any time.

Whom should I contact if I have any questions?

If you have any questions on your participation, please contact Dr Luke Nelson via email luke@chirosolutions.com.au or phone +61395570251.

Yours sincerely

Dr. Luke Nelson, B App Sci (Clin Sci.)/B Chiro Sci, ICSSD, PGrad.Dip.SportChiro, M.Sport.Sci,

Dr. Rick Ames, BSc, DC, FACCS, ICSSD, FACC

Dr. Brett Jarosz, BAppSc(CompMed), MClinChiro, ICSSD, PGradDipSportsChiro, DACNB, FICC

Dr. Henry Pollard BSc, Grad DC, Grad Dip AppSc, MSportSc, PhD, ICSSD,

Dr. Pete Garbutt B.Sc., M.Chiro., M.Chiro.Sports.Sci., ICSSD

Dr Cliff Da Costa, BSc, MSc, MS, PhD

If you have any concerns about your participation in this project, which you do not wish to discuss with the researchers, then you can contact the Ethics Officer, Research Integrity, Governance and Systems, RMIT University, GPO Box 2476V VIC 3001. Tel: (03) 9925 2251 or email human.ethics@rmit.edu.au

----

Do you consent to participate in this study? (If NO, simply close this browser)

◯ Yes

2. Gender

[Please choose]

3. Age (yrs)

[Please choose]

4. How many years have you been in private practice?

[Please choose]

5. Have you undertaken any further university education relevant to the field of Sports Chiropractic?

Please click all relevant

- ☐ No
- ☐ Post graduate certificate
- ☐ ICSSD/ICSSP
- ☐ Post graduate diploma
- ☐ Masters
- ☐ PhD

6. Continent in which you predominantly practice?

Please select

- ○ Asia
- ○ Africa
- ○ Europe
- ○ North America
- ○ South America
- ○ Oceania

Page 03

7. Do you read health care related research?

[Please choose]

8. How many research articles related to the field of Sports Chiropractic would you read per week (on average)?

Number of articles per week

9. How much time would you spend per week (on average) reading research related to the field of Sports Chiropractic?

How much time in hours

10. Do you currently work with a sports team full time?

[Please choose]

11. Do you currently treat athletes involved in professional level sport?

[Please choose]

12. Have you previously treated athletes involved in professional level sport?

[Please choose]

Page 04

13. Do you currently treat athletes involved in semi-professional level sport?

[Please choose]

14. Have you previously treated athletes involved in semi-professional level sport?

[Please choose]

15. Do you currently treat Olympic level athletes?

[Please choose]

16. Have you previously treated Olympic level athletes?

[Please choose]

17. Do you perform manual spinal manipulation/adjustments?

[Please choose]

18. Do you perform manual peripheral manipulation/adjustments?

[Please choose]

19. Do you perform mobilisation?

[Please choose]

20. Do you use low force techniques (i.e. drop piece, activator)?

[Please choose]

21. Do you perform soft tissue therapy?

[Please choose]

22. Do you use dry needling?

[Please choose]

23. Do you perform Instrument Assisted Soft Tissue therapy (i.e Graston, FAKTR, Gua Sha ect.)?

[Please choose]

24. Do you use rigid taping?

[Please choose]

25. Do you use Kinesio-type taping (including other brands)?

[Please choose]

26. Do you use any physiological therapeutics (i.e therapeutic ultrasound, laser ect.)?

[Please choose]

27. Are the MAJORITY (>50%) of your treatments performed multimodal?

[Please choose]

28. Do you treat NON-SPINAL MUSCULOSKELETAL conditions (shoulder, feet, knees ect.)?

[Please choose]

29. What percentage of your patients have a PRIMARY complaint that is NON-SPINAL MUSCULOSKELETAL in nature?

%

30. What percentage of patients do you prescribe rehabilitation exercise to?

%

31. What percentage of patients do you prescribe ergonomic advice to?

%

32. What percentage of patients do you prescribe nutritional advice to?

%

33. Do you REFER to other health practitioners?

[Please choose]

34. How often do you REFER to other health practitioners?

Never	Rarely	Often	Frequently
○	○	○	○

35. Which type of health practitioner do you REFER to?
Please tick all that apply

- ☐ None
- ☐ Orthopedic surgeon
- ☐ Sports Physician
- ☐ General practitioner
- ☐ Physiotherapist/Physical therapist
- ☐ Massage therapist (myotherapist)
- ☐ Athletic trainer
- ☐ Osteopath
- ☐ Podiatrist
- ☐ Strength and conditioning
- ☐ Exercise physiologist
- ☐ Neurosurgeon
- ☐ Neurologist
- ☐ Neurophysiologist
- ☐ Dietitian
- ☐ Nutritionist
- ☐ Naturopath
- ☐ Psychologist
- ☐ Dentist
- ☐ OTHER ____________

36. Do you CO-MANAGE with other health practitioners?

[Please choose]

37. How often do you CO-MANAGE with other health practitioners?

Never	Rarely	Often	Frequently
○	○	○	○

38. Which type of health practitioner do CO-MANAGE with?
Please tick all that apply

- ☐ None
- ☐ Orthopedic surgeon
- ☐ Sports Physician
- ☐ General practitioner
- ☐ Physiotherapist/Physical therapist
- ☐ Massage therapist (myotherapist)
- ☐ Athletic trainer
- ☐ Osteopath
- ☐ Podiatrist
- ☐ Strength and conditioning
- ☐ Exercise physiologist
- ☐ Neurosurgeon
- ☐ Neurologist
- ☐ Neurophysiologist
- ☐ Dietitian
- ☐ Nutritionist
- ☐ Naturopath
- ☐ Psychologist
- ☐ Dentist
- ☐ OTHER ____________

Last Page

Thank you for completing this questionnaire!

We would like to thank you very much for helping us.

Your answers were transmitted, you may close the browser window or tab now.
